# Characteristics and Prognosis of Exercise-Related Sudden Cardiac Arrest

**DOI:** 10.3389/fcvm.2018.00102

**Published:** 2018-07-26

**Authors:** Tomi M. Toukola, Janna P. Kauppila, Lasse Pakanen, Marja-Leena Kortelainen, Matti Martikainen, Heikki V. Huikuri, M. Juhani Junttila

**Affiliations:** ^1^Medical Research Center Oulu, Oulu University Hospital and University of Oulu, Oulu, Finland; ^2^Forensic Medicine Unit, National Institute for Health and Welfare, Oulu, Finland; ^3^Department of Forensic Medicine, Research Unit of Internal Medicine, Medical Research Center Oulu, University of Oulu, Oulu, Finland; ^4^Center for Pre-hospital Emergency Care, Oulu University Hospital, Oulu, Finland

**Keywords:** sudden cardiac arrest, physical activity, resuscitation, survival, initial rhythm

## Abstract

**Introduction:** The previous studies about exercise-related sudden cardiac arrest (SCA) have mainly focused on sports activity, but information related to SCA in other forms of physical exercise is lacking. Our aim was to identify characteristics and prognosis of SCA victims in the general population who suffered SCA during physical activity.

**Methods and results:** We collected retrospectively all cases of attempted resuscitation in Oulu University Hospital Area between 2007 and 2012. A total of 300 cases were of cardiac origin. We only included witnessed cases with Emergency Medical System arrival time ≤15 min. Cases of low-intensity physical activity were excluded. A total of 47 SCAs occurred during moderate-to-vigorous physical activity (exercise-group) and 43 cases took place at rest (rest–group). The subjects in exercise-group were younger compared to the rest-group (60 ± 14 years vs. 67 ± 14 years, *p* = 0.016). The initial rhythm recorded was more often ventricular fibrillation (VF) in exercise-group compared to the rest-group (77 vs. 50%, *p* = 0.010). Pulseless electrical activity (PEA) was rare in exercise-group compared to the rest -group (2.1 vs. 14%, *p* = 0.033, respectively). Bystander cardiopulmonary resuscitation (CPR) was more often performed when SCA took place during physical exercise (47 vs. 23 %, *p* = 0.020). Survival rates to hospital discharge were higher in the exercise-group compared to the rest -group (49 vs. 9.3%, *p* < 0.0001).

**Conclusions:** SCA occurring during physical activity is more frequently a result of VF and bystander CPR is more often performed. There is also a notably better survival rate to hospital discharge.

## Introduction

Regular physical activity has beneficial effects on cardiovascular health (1) and reduces cardiovascular mortality both in primary (2) and secondary prevention (3). However, an increased risk of cardiovascular events, such as acute myocardial infarction and sudden cardiac death (SCD), has been noted during and immediately after intense physical exertion (4–6). Sudden cardiac arrest (SCA) is a potentially reversible event of arrhythmia causing collapse of the cardiovascular system. The prognosis of out-of-hospital SCA has improved during the last decade but the survival to hospital discharge has remained low (7–9). The improvement in survival of out-of-hospital SCA might be due to improved rates of bystander CPR and increased dissemination of automated external defibrillator (AED) as well as improved overall medical care.

In recent years a few studies have shown the low incidence of sports-related SCA in the general population and a better prognosis when cardiac arrest occurs in relation to exercise (10–12). These studies have, however, focused on sports activity and excluded other types of physical activity. For example a study by Wu et al. showed that physical labor was the most common circumstance of exercise-related SCD (13). The exact etiology of SCA was unknown in a significant amount of victims due to lack of systematic investigation with autopsy in case of death. Our aim was to identify characteristics and prognosis of SCA victims in the general population suffering cardiac arrest during moderate-to-vigorous physical activity compared to those at rest.

## Methods

**Figure 1:**
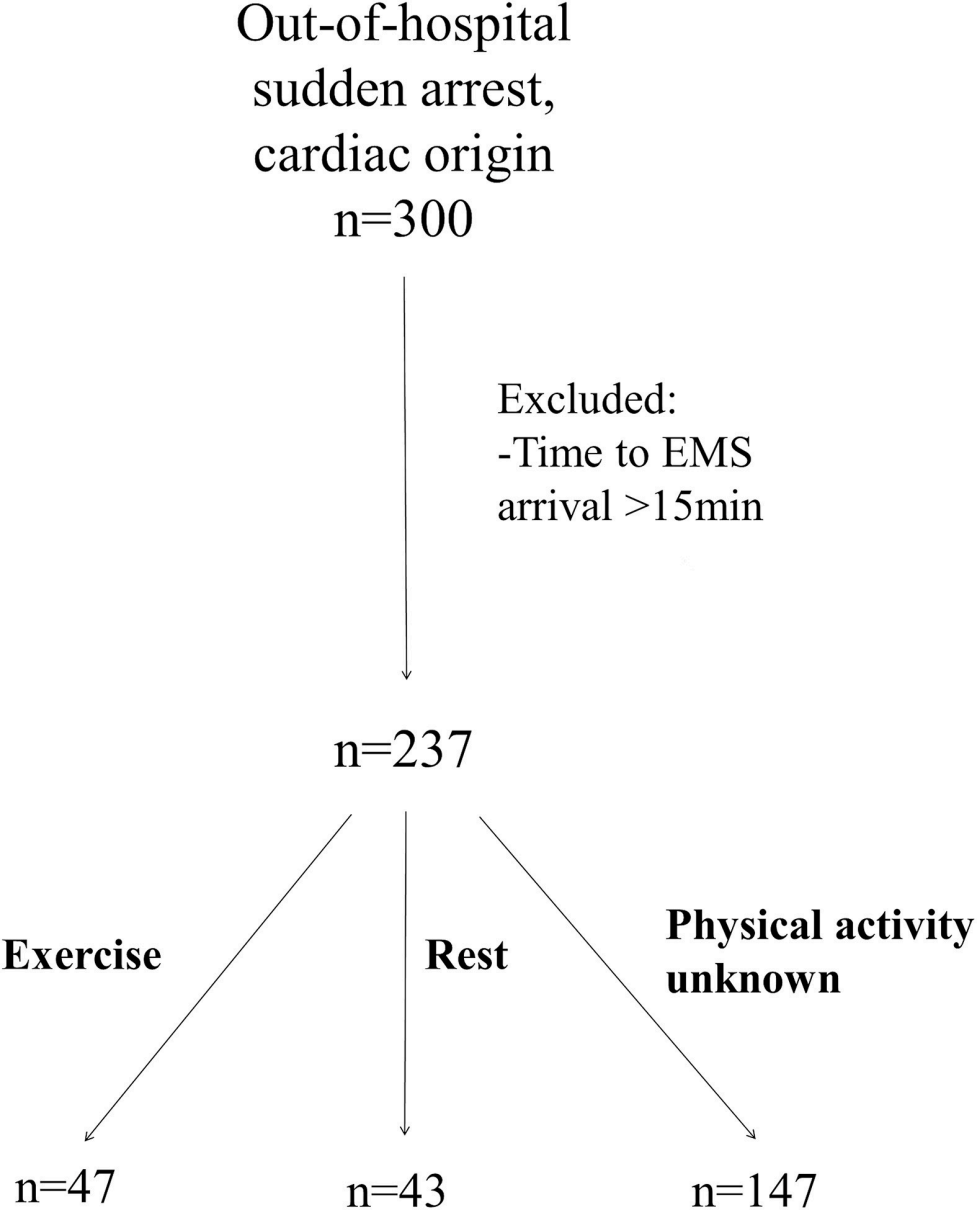
Study populations.

Study population was gathered retrospectively from an emergency service data of Oulu University Hospital between the years 2007 to 2012. Emergency medical services (EMS) in the Oulu University Hospital District are performed with ambulances, medical helicopter, and a unit with an emergency physician on board. Ambulance services can be further divided into first response units, and basic and advanced level ambulances. Data was acquired from out-of-hospital SCA cases where emergency physician was consulted or was at the scene. A total of 300 SCA subjects due to cardiac causes were identified. Utstein recommendation for uniform reporting of cardiac arrest (14) was used in documentation of emergency service data. The data included the initial rhythm monitored by the first-response unit, time delay from the collapse to EMS arrival, information on bystander CPR and description of circumstances related to SCA. Only witnessed cases of SCA were included in the analysis to ensure an accurate estimate of physical activity. To reduce the effect of long time delay to EMS arrival on initial rhythm and survival, we included only cases with up to 15 min between collapse and first-responder arrival. After the exclusions, 237 cases were included in the study (Figure 1).

In cases where cardiac arrest led to sudden death, a comprehensive medico-legal autopsy was conducted to exclude non-cardiac causes. Predefined criteria for diagnosing the underlying structural heart disease were used (15). There were a total of 102 cases of SCD. All subjects with a medico-legal autopsy were originally part of the FinGesture study population, which has been described earlier (15, 16). FinGesture includes consecutive autopsy-verified out-of-hospital victims of SCD in a specific geographical area in northern Finland currently gathered between the years 1998 and 2012. The history of cardiac disease and medications were acquired by reviewing medical records of the victims, post-mortem examination reports, and questionnaires to next of kin. A total of 129 SCA cases were successfully resuscitated and survived to hospital admission. In these cases, the information on prior medications and diagnosed cardiac disease was gathered from medical records in Oulu University Hospital electronic archives. The underlying cause for SCA was sorted out by clinical examinations, including echocardiography and coronary angiography. We only included cases with convincing evidence of cardiac cause.

The level of physical activity at the time of death was assessed according to the information from emergency units, death certificates, and police reports, questionnaires to next of kin, and medical records documented by treating physicians. We only included subjects with SCA during moderate-to-vigorous physical activity and excluded low-intensity activity, and subjects with inadequate information about physical activity. We assessed the metabolic equivalents of different physical activities according to the estimates of Jettè et al. (17) and included activities that are considered of at least 4 metabolic equivalents. SCA was defined to be related to physical activity, when the SCA occurred during or within 1 h after physical activity. SCA was considered to take place at rest when the subject experienced witnessed cardiac arrest while sleeping, sitting or lying down (Figure 1).

The study complies with the Declaration of Helsinki, and the ethics committee of the University of Oulu approved the study. The National Supervisory Authority for Welfare and Health (Valvira) approved the review of post-mortem data by the investigators.

### Statistical analysis

χ2 analyses were used to detect differences in dichotomous variables. Three different initial rhythms were present and therefore pairwise comparisons by χ2, corrected for multiple testing, were performed because significant main effect across the initial rhythms was detected. Age and time to EMS arrival were verified to have Gaussian distribution by skewness test. These continuous variables are reported as mean (standard deviation). Odds ratio and its 95% confidence interval (CI) for survival to hospital discharge was calculated by multivariate logistic regression analysis and adjusted for age, gender, bystander CPR, initial rhythm recorded, and prior diagnosed cardiac disease. The Statistical Package for Social Studies 21 (SPSS Inc., Chicago, IL) was used to perform the analyses and a *p*-value < 0.05 was considered statistically significant.

## Results

After exclusions, a total of 237 victims of SCA were included in the study. Of these subjects, 36 (15%) SCA occurred during moderate-to-vigorous physical activity and in 11 (4.6%) within a 1-h time frame after the cessation of physical exercise (exercise –group). In 43 (18%) subjects SCA took place at rest (rest –group) and 147 (62%) of cases were further excluded while the circumstance at the time of SCA did not meet the criteria of either rest or exercise or the level of activity could not be accurately assessed. The types of exertion related to SCA are presented in Table 1. Cycling (26%) and heavy labor (21%) were the most common types of exertion related to SCA.

**Table 1:** Distribution of different types of physical activity related to sudden cardiac arrest.

**Type of physical activity**	**Number of subjects (*n* = 47)**	**Percentage(%)**
Cycling	12	26
Heavy labor	10	21
Snow shoveling	4	8.5
Skiing	3	6.4
Walking/jogging	3	6.4
Ball games	3	6.4
Dancing	2	4.3
Swimming	2	4.3
Intercourse	1	2.1
Other	7	15

Subject characteristics are presented in Table 2. Subjects in exercise–group were younger compared to the rest–group (60 ± 14 years vs. 67 ± 14 years, *p* = 0.016, respectively). Male gender was somewhat more common in the exercise–group although this finding did not reach statistical significance (*p* = 0.094). The overall incidence of prior diagnosed cardiovascular disease was less common in exercise–group compared to the rest–group (54 vs. 76%, *p* = 0.038, respectively). Subjects in the rest–group were far more likely to have prior diagnosed congestive heart failure compared to those in exercise–group (24 vs. 2.1%, *p* = 0.002, respectively). Also prior diagnosis of CAD was more common in the rest -group, but this finding was not statistically significant (*p* = 0.14). Prior diagnosis of hypertension was less common in the exercise–group (30 vs. 55%, *p* = 0.017). No differences could be found in the proportion of either prior diagnosis of hyperlipidemia or diabetes mellitus. The distribution of ischemic cause of SCA was fairly similar between the two groups (exercise–group: 89% vs. rest–group: 81%, *p* = 0.28).

**Table 2:** Characteristics of subjects of sudden cardiac arrest in exercise-group and rest-group.

**Variable**	**Exercise-related SCA (*n* = 47)**	**SCA at rest (n = 43)**	***P*-value**
Age, years (*SD*)	60(14)	67 (14)	**0.016**
Male gender	92% (43)	79 % (34)	0.094
Time to EMS arrival, min (*SD*)	7.1(5.0)	9.0 (4.3)	0.065
Prior diagnosed CAD	17% (8)	30 % (13)	0.14
Prior congestive heart failure	2.1% (1)	24 % (10)	**0.002**
Prior cardiac disease	40% (19)	63 % (27)	**0.034**
Prior hypertension	30% (14)	55 % (24)	**0.017**
Prior dyslipidemia	17% (8)	19 % (8)	0.80
Diabetes Mellitus	15% (7)	26 % (11)	0.19
SCA due to ischemic heart disease	89% (42)	81% (35)	0.28

*CAD, Coronary Artery Disease; EMS, Emergency Medical Service; SCA, Sudden Cardiac Arrest; SD, Standard Deviation. Statistically significant p-values (< 0.05) are highlighted as bold values*.

Resuscitation and survival information for exercise-group and rest-group are displayed in Table 3. Survival of SCA to hospital discharge was multiple times higher in exercise–group compared to the at-rest–group (49 vs. 9.3%, *p* < 0.0001) and the risk of death before hospital discharge was 9.3-fold (2.9–30, *p* = 0.00020) when SCA occurred at rest compared to exercise-related SCA. When adjusted for age, gender, bystander CPR, initial rhythm recorded, and prior diagnosed cardiac disease in a multivariate regression analysis, the risk of death was still 7.0 (1.4–35, *p* = 0.019) among those dying at rest. The difference between the two groups was evident in subjects under 65 years of age (exercise–group: 50% vs. rest–group: 13%, *p* = 0.005) and over the age of 65 years (exercise –group: 47% vs. rest –group: 5.3%, *p* = 0.003). The mean time to EMS arrival was 1.9 min shorter in exercise–group, but this finding did not reach statistical significance (exercise–group: 7.1 min ±5.0 vs. rest–group: 9.0 min ±4.3, *p* = 0.065). Subjects in exercise-group had more often a shockable initial rhythm compared to rest–group (77 vs. 50%, *p* = 0.009, respectively). Additionally PEA was notably more common in rest–group compared to exercise–group (14 vs. 2.1%, *p* = 0.033, respectively). Ventricular fibrillation was the initial rhythm in all cases of shockable rhythm both in exercise -group and rest-group. The rate of bystander CPR was significantly lower in the rest -group compared to the exercise -group (23 vs. 47%, *p* = 0.020, respectively).

**Table 3:** Resuscitation and survival information among victims of SCA during exercise and at rest.

**Variable**	**Exercise-related SCA (*n* = 47)**	**SCA at rest (*n* = 43)**	***P*-value**
Initial rhythm recorded			**0.016** ^*^
VF	77% (36)	50% (22)	
Asystole	21% (10)	36% (15)	
PEA	2.1% (1)	14% (6)	
Initial rhythm recorded			**0.009**
Shockable	77% (36)	50% (22)	
Non-shockable	23% (11)	50% (21)	
Bystander CPR	47% (22)	23% (10)	**0.020**
Survival to hospital discharge	49% (23)	9.3% (4)	**<0.0001**
Age < 65 years	50% (14)	13% (3)	**0.005**
Age ≥ 65 years	47% (9)	5.3% (1)	**0.003**

* *Main effect across the groups. CPR, Cardiopulmonary Resuscitation; PEA, Pulseless Electrical Activity; SCA, Sudden Cardiac Arrest; VF, Ventricular Fibrillation. Statistically significant p-values (< 0.05) are highlighted as bold values*.

## Discussion

In this prospective study, we had 47 subjects in whom SCA occurred during or immediately after physical exercise over our 6-year study period in a study area with 409,938 inhabitants. Therefore, the overall incidence of exercise-related SCA was 1.9 per 100,000 person-years in our study area. In previous studies the incidence of exercise-related SCA has varied between 0.6 and 2.1 per 100 000 person-years (10–12). The definition of exercise has, however, varied between the studies. These studies have included mainly sports activities, which do not take into account the effect of heavy physical labor. In a Chinese study, physical labor was the most common type of exertion related to SCD (13). The strength of our study lies in the fact that our study subjects are from all walks of life and we took into account a broad variety of different types of exercise. Exercise-related SCAs in the general population occur in older and less fit subjects compared to studies with athletes as study subjects (5, 18, 19). Therefore, the risk of potential hazards of physical exercise is higher in the general population. In our study, cycling was found to be the most common type of activity related to SCA, followed by heavy labor. In Spain cycling was the most common trigger of exercise-related SCD (20). Snow shoveling and skiing were also relatively common circumstances related to exercise-related SCA and this finding attributes to the northern geographic location of our study population. Our previous study about exercise-related SCD had a fairly similar finding (21). The most common types of physical activity in our study are not usually performed in public sporting facilities. Thus, the dissemination of AED to public facilities would likely have no beneficial effect on these types of exercise-related SCA. We did not have information about bystander usage of AED in our data.

Subjects who suffered SCA in relation to physical exercise were younger. This finding has been similar in previous studies (10, 12). Younger subjects are more likely to perform physical activity and are more likely to achieve moderate-to-vigorous intensity, which might explain the age difference. In a study by Marijon et al. significant difference in mean ages between sports-associated and non-sports-associated SCA was not detected, but only subjects between the ages 35 and 65 were included (11). Male gender was somewhat more frequent among exercise-related SCA, but this finding was not statistically significant. Numerous previous studies have shown male dominance in exercise-related cardiac events (10, 18, 22). Time to EMS arrival took on average 1.9 min longer when SCA occurred at rest, this finding did not, however, reach statistical significance. Subjects with SCA at rest had more often prior congestive heart failure, diagnosed CAD or any other prior cardiac disease. Prior diagnosis of hypertension was also more common in the rest–group. Especially congestive heart failure might reduce overall time spent physically active, and therefore make exercise-related SCA unlikely. On the other hand, sedentary subjects have the highest transient risk of adverse cardiac event during exercise (4–6). In our study we did not have information about the habitual physical activity or fitness level of subjects. The cause of SCA was mostly due to ischemic origin in both groups and no significant difference could be found. However, only 17% had a previously made diagnosis of CAD in the exercise-group highlighting the importance of occult ischemic heart disease as the substrate for SCA. It is important to find and treat subjects in the general population with undiagnosed CAD to prevent exercise-related SCA and SCA in general. A comprehensive medico-legal autopsy was performed to all SCA subjects who died before reaching hospital. This approach is more accurate than clinical evaluation alone. The information on autopsy-findings related to exercise-related SCD has been published previously (21).

Initial rhythm recorded by the first EMS unit was shockable in 77% of exercise-related SCAs and 50% in the at-rest-group. This finding is fairly similar compared to previous studies (10–12). In our current study, when the initial rhythm was non-shockable, it was more often asystole than PEA. PEA was the initial rhythm in only 2.1% of exercise-related cases. The proportion of PEA was almost 7-fold in the rest-group and this finding was statistically significant. Bystander CPR was initiated more likely when SCA took place in relation to physical exercise, which has been noted in previous studies (10, 11). Although, in a study by Berdowski et al. a substantially higher rates of bystander CPR were noted (10). Bystander CPR was performed in our study group only in 23% of cases in the rest-group and in 47% of exercise-related cases. In Denmark, targeted national initiatives have been able to increase the rates of bystander CPR and survival rates of out-of-hospital SCA during a 10-year study period (23). Bystander CPR has been shown to improve survival and long-term outcome in SCA (8, 23, 24). All our cases were witnessed and therefore a bystander CPR rate of 23 and 47% is low. Further national initiations should probably be established to improve the abilities of citizens to act in SCA cases. For example, resuscitation training offered in learning institutions has been shown to increase the likelihood of bystander CPR (23).

Survival to hospital discharge was clearly higher among exercise-related SCA. Berdowski et al. presented a similar finding in subjects over the age of 35 and the effect was more evident in the oldest (age over 65 years) (10). Another study by Marijon et al. did not find evidence of higher survival in the setting of sports activity when adjusted for resuscitation variables (11). We had, however, a similar difference between the two study groups in survival to hospital discharge both in subjects under and over the age of 65 years. The risk of death was still 7.0-fold among those with SCA at rest, even after adjustments for age, gender, bystander CPR, initial rhythm recorded and prior diagnosed cardiac disease in a multivariate regression analysis. There are probably several reasons for this result. Subjects with SCA during rest might have had a more severe prior cardiac disease. Congestive heart failure was also clearly a more common finding among those with SCA at rest. Regular physical activity has widespread cardioprotective effects. Subjects who experienced SCA in association to physical activity might have been in better shape and therefore possessed a higher probability of survival. This is, however, speculative, because we did not have information about the habitual physical activity and fitness levels of subjects.

## Limitations

The strengths of our study are the high rate of medico-legal autopsies in Finland, available comprehensive medical records, clear difference between physical activity and rest and the fact that we only included witnessed SCAs. However, a few limitations can be identified in our study. The exact subject-specific intensity of physical activity at the time of SCA is difficult to assess prospectively even in witnessed SCAs. There might be some inaccuracy in classification between low-intensity and moderate-intensity activity. The absolute numbers of SCA during exercise and at rest were fairly small in our study and this diminishes the ability to extrapolate the presented findings. The data collected during our study period between the years 2007 and 2012 might not include all resuscitations during that time, so we cannot draw conclusions about overall incidence of exercise-related SCA. We also lacked information about the habitual physical activity and fitness level of subjects.

## Conclusion

The present findings show the relatively good survival rates of exercise-related SCA in the general population. Subjects who suffer SCA in association with physical activity tend to be younger and previously healthier and the initial rhythm is more often shockable. Cycling and heavy physical labor were the most common types of physical activity related to SCA. The rate of bystander CPR should be improved.

## Author contributions

TT and MJ designed the study. M-LK and LP contributed significantly by performing the medico-legal autopsies between the years 2007 and 2012. JK gathered the resuscitation data. TT executed the analyses. HH and MJ contributed expertise in cardiology and substantial scientific input in the interpretation of the results. MM contributed expertise in emergency medicine. TT drafted the manuscript and all authors took part in manuscript modifications. The final version was accepted by all authors.

### Conflict of interest statement

The authors declare that the research was conducted in the absence of any commercial or financial relationships that could be construed as a potential conflict of interest.
